# Cornu Cutaneum: Case Reports of Patients With a Cutaneous Horn Associated With Either a Verruca Vulgaris or an Inverted Follicular Keratosis and a Review of the Etiologies of Cutaneous Horns

**DOI:** 10.7759/cureus.46747

**Published:** 2023-10-09

**Authors:** Philip R Cohen

**Affiliations:** 1 Dermatology, Davis Medical Center, University of California, Sacramento, USA; 2 Dermatology, Touro College of Osteopathic Medicine, Vallejo, USA

**Keywords:** verruca, wart, seborrheic, keratosis, inverted follicular keratosis, horn, cutaneum, cutaneous, cornu, cancer

## Abstract

A cutaneous horn, referred to as a cornu cutaneum in Latin, presents as a mound of keratinizing epithelium. The etiology of the cutaneous horn is associated with the lesion at its base. In addition to numerous benign and malignant neoplasms, cutaneous horns may be related to infections and skin conditions. The features of a 22-year-old woman with a cutaneous horn associated with a recalcitrant verruca vulgaris on her left fifth toe are described. In addition, the characteristics of a 57-year-old man with an inverted follicular keratosis-related cutaneous horn on his upper lip are reported. In order of decreasing frequency, a cutaneous horn is most associated with either an actinic keratosis (25%), a squamous cell carcinoma (19%), a seborrheic keratosis (19%-20%), or a verruca vulgaris (18%). Adnexal neoplasms, epithelial lesions, fibrous lesions, granular cell tumors, hamartomas, histiocytic lesions, melanocytic nevus, premalignant keratoses, a subungual lesion, and vascular lesions comprise the benign neoplasms that have been observed at the base of a cutaneous horn. Dermatologic conditions that have been associated with a cutaneous horn include discoid lupus erythematosus (three patients) and one patient with either palmoplantar keratoderma, psoriasis, or sarcoidosis. Human papillomavirus infection presenting as a verruca vulgaris is the most commonly associated infection; pox virus-related molluscum contagiosum is another viral infection that is less often observed associated with a cutaneous horn. Leishmaniasis, rhinosporidiosis, and cutaneous tuberculosis are rare cutaneous horn-related infections. A malignant tumor-associated cutaneous horn is most frequently caused by squamous cell carcinoma; other less common cancers include basal cell carcinoma, sebaceous carcinoma, verrucous carcinoma, and malignant melanoma. A cancer-related cutaneous horn has only been described in two patients with Kaposi sarcoma and one patient with either Merkel cell carcinoma or Paget disease of the breast or metastatic renal cell carcinoma. In summary, a cutaneous horn is potentially related to a tumor, an infection, or a skin disorder; an adequate evaluation of the base of the cutaneous horn is usually required to establish the associated diagnosis.

## Introduction

Cornu cutaneum is the Latin term for what is commonly referred to as a cutaneous horn. A cutaneous horn consists of a mound of keratinizing stratum corneum. Benign and malignant tumors, infections, and cutaneous conditions can be associated with a cutaneous horn [1-20].

A cutaneous horn is a clinical diagnosis; its height is larger than half of its greatest diameter. Giant cutaneous horns can develop. Evaluating the epidermis and dermis of the skin at the base of the horn is often necessary to determine the diagnosis [1-5,11].

A woman with a verruca vulgaris-associated cutaneous horn on her right great toe for which the viral infection was recalcitrant to therapy is described. In addition, a man with an inverted follicular keratosis-related cutaneous horn, almost completely removed by the biopsy, without recurrence is reported. The neoplasms, infections, and skin disorders that can be associated with a cutaneous horn are reviewed [1-20].

## Case presentation

Case 1

**Figure 1:**
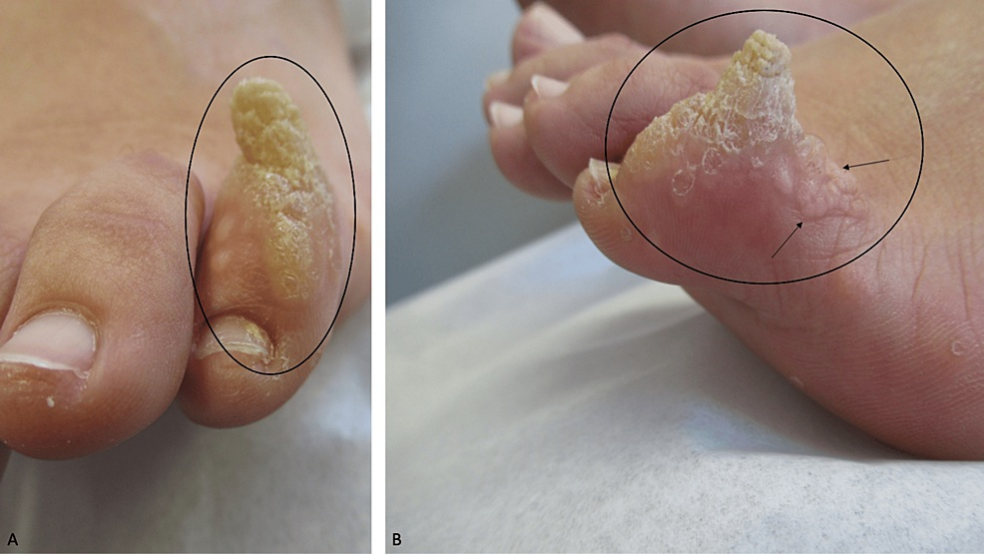
A cutaneous horn associated with verruca vulgaris. (A) Frontal and (B) lateral views of the 35 mm × 15 mm verrucous nodule with a large cutaneous horn (within the black oval) on top of the left fifth toe of a 22-year-old woman. Typical-appearing verrucous papules of verruca vulgaris (black arrows) are adjacent to the cutaneous horn.

A 22-year-old healthy woman presented for evaluation of a 35 mm × 15 mm verrucous nodule with a large cutaneous horn on top of the left fifth toe of three years duration (Figure 1). It had previously been treated with cryotherapy using liquid nitrogen. She was only able to wear open sandals. She had difficulty wearing socks and could not wear shoes or sneakers.

**Figure 2:**
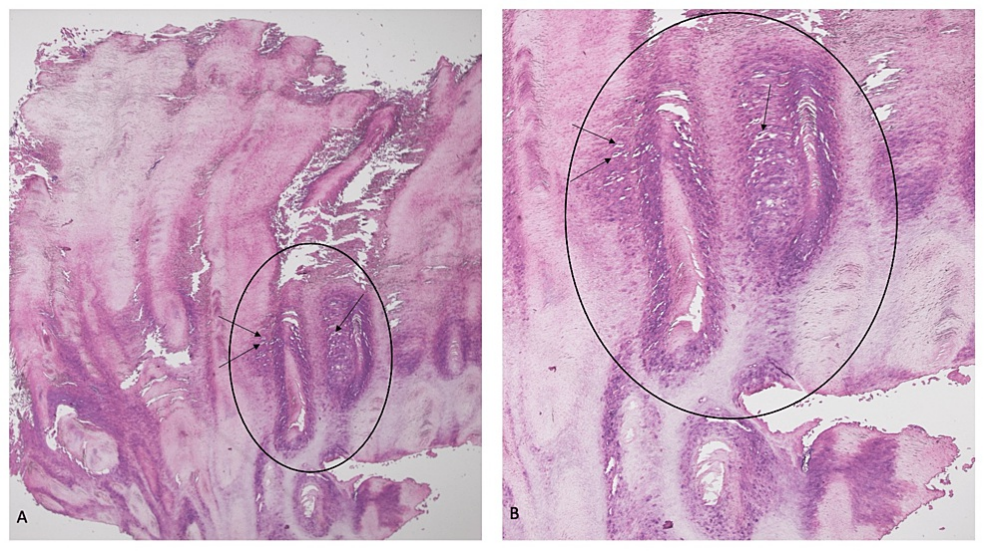
Microscopic features of a verruca vulgaris-associated cutaneous horn. (A) Low- and (B) high-magnification views of the cutaneous horn show massive hyperkeratosis and focal parakeratosis. Features of verruca vulgaris, including hypergranulosis (within black oval) and koilocytes (black arrows) are also present. Hematoxylin and eosin: (A) ×4; (B) ×10.

Microscopic examination of the biopsied specimen revealed massive hyperkeratosis consisting of cornified epithelium with focal parakeratosis; there was hypergranulosis and occasional koilocytes (Figure 2). The pathology showed a cutaneous horn and the surface of a verruca.

Correlation of the clinical presentation and pathologic findings established the diagnosis of the verruca vulgaris-associated cutaneous horn. Several interventions were attempted for the management of the cutaneous horn-associated wart. Unsuccessful treatments included topical 40% urea cream, immunotherapy with topical imiquimod 5% cream each evening, monthly intralesional injections of Candida antigen, and topical squaric acid.

Case 2

**Figure 3:**
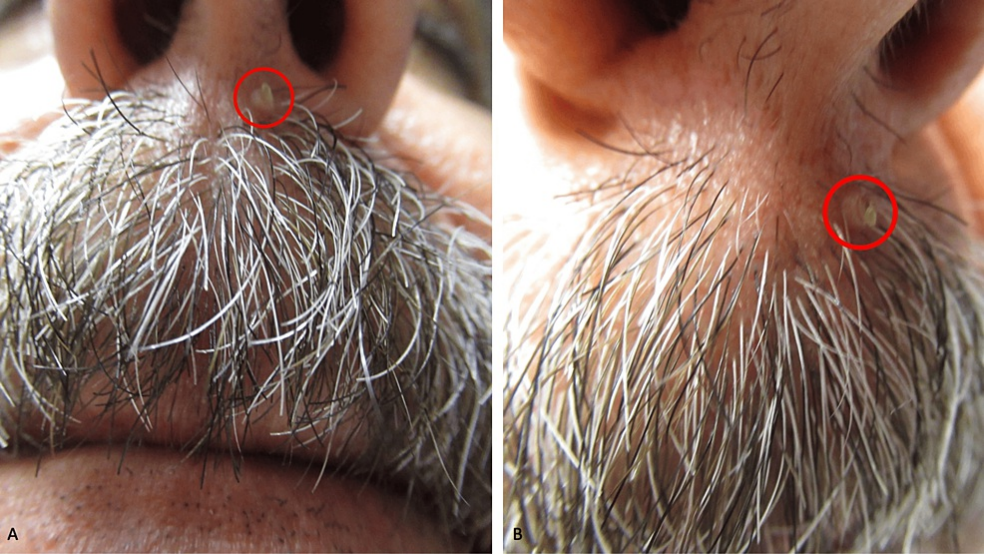
A cutaneous horn associated with an inverted follicular keratosis. (A) Distant and (B) closer views of the cutaneous horn overlying the 2 mm × 2 mm papule (within the red oval) on the left side of the upper lip beneath the nostril of the 57-year-old man.

A 57-year-old healthy man presented for evaluation of a new lesion on the left side of his upper lip. A cutaneous exam revealed a 2 mm × 2 mm papule with an overlying cutaneous horn on the left side of his upper lip beneath his nostril (Figure 3). A shave biopsy was performed. 

**Figure 4:**
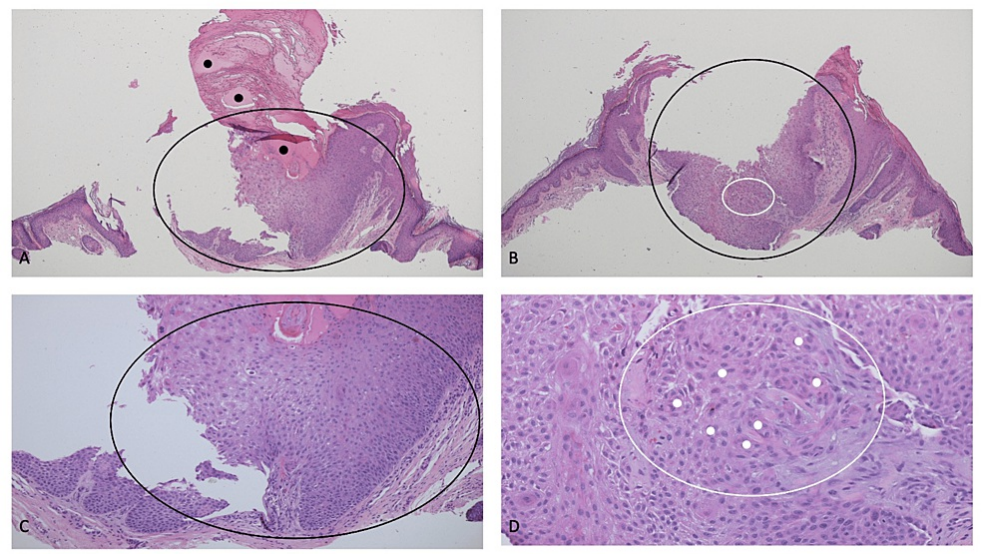
Microscopic features of an inverted follicular keratosis-associated cutaneous horn. (A and B) Distant and (C and D) closer views of the cutaneous horn overlying the inverted follicular keratosis. The biopsy specimen was bisected into two pieces during processing. Half of the specimen is shown in (A) and (C), and the other half of the specimen is shown in (B) and (D). The cutaneous horn, which contains hemorrhage (black circles), is composed of parakeratotic keratin (A). The inverted follicular keratosis is an endophytic nodular lesion that extends into the dermis (within the black ovals). Some of the squamous eddies are demonstrated within the white ovals; they consist of central whorls of mature squamous cells (white circles). Hematoxylin and eosin: (A) ×4; (B) ×4; (C) ×10; (D) ×20.

Microscopic examination of the tissue specimen showed an endophytic lesion composed of benign epithelial cells showing numerous squamous eddies that extended into the underlying dermis; this was an inverted follicular keratosis, which focally extended to the base of the specimen (Figure 4). In addition, there was a horn-like mass of parakeratotic keratin, which contained hemorrhage, overlying the lesion; this represented a cutaneous horn.

Correlation of the clinical morphology and the pathologic finding established a diagnosis of inverted follicular keratosis associated with an overlying cutaneous horn. No additional treatment was required. A follow-up examination two months later showed complete healing of the biopsy site without recurrence.

## Discussion

A cutaneous horn has been associated not only with numerous benign neoplasms but also with several malignant tumors [1-5,14-20]. The etiology may be related to an underlying cutaneous infection [1-5,7-10]. In addition, albeit rare, a cutaneous horn may be a clinical manifestation of a skin disease [10-13].

Several retrospective studies have been performed on cutaneous horns. Some of the studies are summarized in Table 1 [1-4]. The most associated lesions with a cutaneous horn are actinic keratosis, squamous cell carcinoma, seborrheic keratosis, and verruca vulgaris. Several benign tumors have been associated with a cutaneous horn (Table 2) [1-5]. These include adnexal neoplasms, epithelial lesions, fibrous lesions, granular cell tumors, hamartomas, histiocytic lesions, melanocytic nevus, premalignant keratoses, subungual lesions, and vascular lesions [1-5].

**Table 1:** Summary of retrospective studies of cutaneous horns. ^a^Squamous cell carcinomas also include squamous cell carcinomas in situ (which are also referred to as Bowen’s disease) and keratoacanthoma. ^b^These include one benign lichenoid keratosis. ^c^The investigators include basosquamous papilloma (at least one) and inverted follicular keratosis (at least one) in the 14 lesions that they categorize as seborrheic keratoses. AK, actinic keratosis; BCC, basal cell carcinoma; IFK, inverted follicular keratosis; SCC, squamous cell carcinoma; SK, seborrheic keratosis; Refs, references; VV, verruca vulgaris; # number

Author	Number of cutaneous horns in study	AK # (%)	SCC^a^ # (%)	SK # (%)	VV # (%)	IFK # (%)	BCC # (%)	Refs
Yu et al.	643	123 (19)	130 (20)	135 (21)	100 (16)	0 (0)	0 (0)	[4]
Schosser et al.	230	86 (37)	44 (19)	38^b^ (17)	52 (23)	0 (0)	3 (1.3)	[3]
Mehregan	100	35 (35)	11 (11)	17 (17)	30 (30)	4 (4)	0 (0)	[1]
Bart et al.	37	8 (22)	8 (22)	1-12^c^ (3-35)	0 (0)	1-12^c ^(3-35)	0 (0)	[2]
Total	1010	252 (25)	193 (19)	191-202 (19-20)	182 (18)	5-16 (0.5-1.6)	3 (0.3)	[1-4]

**Table 2:** Benign skin lesions associated with cutaneous horns.

Skin lesions	References
Epithelial	[1-5]
Benign hyperplastic epithelioma	[1-5]
Benign lichenoid keratosis	[1-5]
Clear cell acanthoma	[1-5]
Epidermolytic acanthoma	[1-5]
Porokeratosis of Mibelli	[1-5]
Prurigo nodularis	[1-5]
Pseudoepitheliomatous hyperplasia	[1-5]
Seborrheic keratosis	[1-5]
Squamous papilloma	[1-5]
Fibrous	[1-5]
Cicatrix-burn	[1-5]
Cicatrix-traumatic	[1-5]
Digital fibrokeratoma	[1-5]
Fibroepithelial polyp	[1-5]
Fibroma	[1-5]
Follicular	[1-5]
Epidermal inclusion cyst	[1-5]
Inverted follicular keratosis	[1-5]
Pilar cyst	[1-5]
Pilomatricoma	[1-5]
Trichilemmal horn	[1-5]
Tricholemmoma	[1-5]
Granular cell	[1-5]
Granular cell tumor	[1-5]
Hamartoma	[1-5]
Epidermal nevus	[1-5]
Nevus sebaceous	[1-5]
Organoid nevus	[1-5]
Verrucous epidermal nevus	[1-5]
Histiocytic	[1-5]
Dermatofibroma	[1-5]
Juvenile xanthogranuloma	[1-5]
Melanocytic	[1-5]
Intradermal nevus	[1-5]
Miscellaneous	[1-5]
Chalazion	[1-5]
Chronic irritation	[1-5]
Dermoid cyst	[1-5]
Keratotic and micaceous pseudoepitheliomatous balanitis	[1-5]
Radiodermatitis	[1-5]
Subepidermal calcified nodule	[1-5]
Premalignant	[1-5]
Actinic keratosis	[1-5]
Arsenical keratosis	[1-5]
Sebaceous	[1-5]
Sebaceous adenoma	[1-5]
Sebaceous hyperplasia	[1-5]
Subungual	[1-5]
Onycholemmal horn	[1-5]
Vascular	[1-5]
Angiokeratoma	[1-5]
Angioma	[1-5]
Pyogenic granuloma	[1-5]

An inverted follicular keratosis is a benign endophytic follicular tumor with squamous eddies; the latter feature is observed in microscopic evaluation of the lesion and appears as central whorls of the mature squamous cells. Three pathologic patterns of an inverted follicular keratosis have been described: keratoacanthoma-like, filiform or wart-like, and solid or nodular. A retrospective study of 100 cases of inverted follicular keratoses by a dermatopathologist established an incidence of one per 1,400 specimens [6].

Inverted follicular keratoses usually occur in adults; 80% of the patients were aged over 40 years. They occurred twice as often in men than in women. Before evaluation, the lesions had been present ranging from six to eight weeks up to several years. An inverted follicular keratosis typically appears as an asymptomatic, solitary, smooth, or verrucous papule [6].

An inverted follicular keratoses is predominantly located (90%) on the head and neck; 50% of the lesions occur on the face. Similar to the man in this report, an inverted follicular keratosis has previously been observed associated with a cutaneous horn [1,5,6].

In the retrospective study of 100 inverted follicular keratoses, only six of the clinicians considered the correct diagnosis when they were performing the biopsy of the lesion; the most commonly submitted diagnoses were verruca vulgaris (34%), basal cell carcinoma (2%), and seborrheic keratosis (9%) [6]. Similar to most patients with an inverted follicular keratosis-associated cutaneous horn, the correct diagnosis was not entertained during the initial evaluation of the man described in this report. Indeed, the clinical diagnoses suggested to the pathologist were an adnexal neoplasm or a verruca vulgaris. After the biopsy, the site healed nicely, and there was no recurrence of the lesion.

A cutaneous horn has been observed in patients with cutaneous diseases such as chronic cutaneous lupus erythematosus (Table 3) [10-13]. Other dermatologic conditions that have rarely been associated with a cutaneous horn are palmoplantar keratoderma and psoriasis [11,12]. Sarcoidosis of the skin can present with various morphologies; a cutaneous horn on the antitragus was observed as one of the dermatologic manifestations of cutaneous sarcoidosis in a woman who concurrently had other skin lesions of cutaneous sarcoidosis that appeared as firm flesh-colored papules surrounding her left medial canthus and a pink scaly plaque on her posterior neck [13].

**Table 3:** Dermatologic conditions associated with cutaneous horns. DNA, deoxyribonucleic acid; Refs, references

Condition	Comments	Refs
Discoid lupus erythematosus (DLE)	DLE is a subtype of chronic cutaneous lupus erythematosus. At least three patients have been described with DLE presenting as a cutaneous horn. The youngest patient was a 9-year-old boy with generalized DLE with cutaneous horns; he had multiple grouped cutaneous horns ranging in height from 3 mm to 2 cm on his entire body, including the scalp; his mucosal surfaces were spared. Microscopic evaluation demonstrated classical changes of DLE (follicular plugging, epidermal atrophy, vacuolar alteration of the basal cell layer, pigmentary incontinence, and lymphocytic infiltration in a perivascular, periappendageal, and subepidermal location); his anti-nuclear antibody was low (1:160) and anti-double stranded DNA was negative. A topical mid-potency corticosteroid cream (mometasone cream) was applied to the prior cutaneous horn sites after they were removed by shave excision. The second patient was a 28-year-old woman with systemic lupus erythematosus and DLE; she presented with a single 8-cm-long (and 2-cm-long diameter) symptomatic golden-yellow, very thick, crusted, hairy cutaneous horn projecting from her scalp. An excisional biopsy was performed; two specimens were evaluated. The specimen from the base of the lesion had characteristic pathologic features of DLE (vacuolar basal degeneration, superficial perivascular, perifollicular, and perieccrine lymphoplasmacytic infiltration with atrophy of the epidermis). The specimen from the horn showed lamellar hyperkeratosis with focal parakeratosis. There was no recurrence of the lesion after three years of follow-up. The third patient was a 39-year-old white man with a seven-year history of an atrophic plaque of DLE on the left nasal bridge and malar face. He eventually developed a gigantic cutaneous horn measuring 3 to 4 cm in the longstanding plaque of chronic DLE of the nose. Eventually, the cutaneous horn was removed surgically, and the repair incorporated skin grafting. Microscopic examination of the surgical specimen showed typical features of DLE (epidermal atrophy alternating with acanthosis, liquefaction degeneration of the basal cell layer, follicular plugging, and periappendageal lymphocytic aggregates).	[10]
Keratoderma	Palmoplantar keratoderma can be hereditary or acquired. A 55-year-old male bachelor, who worked as a manual laborer, presented with a 35-year history of striate verrucous keratoderma, which was hindering his daily activities. There was not only diffuse involvement of his soles but also extensive involvement - that appeared linearly - on his palms and extending to his fingers. Many of the lesions appeared as cutaneous horns; the biopsy of a heel lesion showed benign hyperplastic epithelium with marked hyperkeratosis and irregular elongation of the rete ridges.	[11]
Psoriasis	A 49-year-old white woman had lesions consistent with psoriasis on her elbows and knees - superficial scaling erythematous plaques - for several years. Two years before the presentation, she was diagnosed with hypothyroidism; however, she did not take her thyroid supplement regularly. Five months before evaluation she developed symptoms of hypothyroidism; in addition to the plaques on her elbows and knees, she also developed numerous irregular horn-like growths that were located distal to the knees. Her laboratory studies were consistent with hypothyroidism. A biopsy of the cutaneous horn-like lesions showed changes of psoriasis: psoriasiform hyperplasia with Munro microabscesses and patchy parakeratosis. A biopsy of the noninvolved skin was consistent with myxedema; there was both edema and mucin in the dermis; the latter was confirmed by the colloidal iron stain. Two months after starting regular thyroid replacement (50 microg of L-thyroxine daily), her thyroid function tests were normal, and no cutaneous horns remained; she only had a few erythematous scaly plaques on her elbows and knees.	[12]
Sarcoidosis	A 53-year-old woman presented with shortness of breath and a tender hyperkeratotic cutaneous horn that was a cone-shaped papule with mild erythema on the right antitragus of two months duration. In addition, she had other cutaneous lesions both on the posterior neck (a pink scaly plaque) and surrounding her left medial canthus (firm flesh-colored papules). Both the antitragus cutaneous horn and neck plaque showed noncaseating granulomas in the superficial dermis; the horn also showed a mound of hyperkeratosis. Fungal and mycobacterial stains were negative, supporting the diagnosis of cutaneous sarcoidosis. Her angiotensin-converting enzyme level was elevated (139 units per liter; normal, 8 to 53 units per liter).	[13]

Cutaneous lesions of fungal-like, mycobacterial, protozoan, and viral infections have been associated with cutaneous horns (Table 4) [1-5,7-10]. Verruca vulgaris is the most associated lesions with the cutaneous horn [1-5]. The woman in this report had typical-appearing verrucous papules adjacent to the cutaneous horn; therefore, verruca vulgaris was suspected to be the diagnosis of her cutaneous horn.

**Table 4:** Infections associated with cutaneous horns. cGY, centigray; HIV, human immunodeficiency virus; HPV, human papillomavirus; MeV, megaelectron volts; MV, megavoltage; NOS, not otherwise specified; Refs, references; X, X-ray

Infection	Comments	Refs
Leishmaniasis	A 31-year-old man - six months earlier - had visited the Dead Sea area near Jericho. He presented with a cutaneous horn that had appeared two months earlier on a Leishmania tropica-associated ulcer on his left mandible. The horn was surgically removed, and microscopic evaluation only showed hyperkeratosis, acanthosis, and papillomatosis. He subsequently presented for additional evaluation, he had several ulcers, including the site of the prior cutaneous horn, which measured from 1 to 4 cm in diameter, and that were located on his right elbow, left leg, and left lower lip (angle of left mandible). Leishman-Donovan bodies were demonstrated from the smear of the ulcers.	[7]
Molluscum contagiosum	Molluscum contagiosum is caused by a pox virus infection. Molluscum contagiosum-related cutaneous horns have been observed not only in HIV-seronegative individuals but also in HIV-seropositive children and adults.	[5]
Rhinosporidiosis	Rhinosporidiosis is considered a fungus-like parasite of the eukaryotic group Mesomycetozoea. It is caused by Rhinosporidium seeberi. The skin lesions of at least two patients with cutaneous rhinosporidiosis have presented with a cutaneous horn. The first patient was a 45-year-old man from India, a farmer who swam in village ponds, had a six-year history of multiple reddish lesions in his nose. He had an oval reddish granulomatous growth with hemorrhagic crusting on the nose, an ulcerated nodule on the left forearm, a crusted verrucous plaque on the left arm, and two small furunculoid lesions discharging pus on the back, and a single 8-mm cutaneous horn on his chest; biopsy of the horn showed hyperplastic epithelium with numerous globular cysts. A systemic workup demonstrated cystic lesions in his lungs; his final diagnosis was nasal rhinosporidiosis with cutaneous and systemic dissemination. All of skin lesions were excised, and he was started on 100 mg of dapsone per day. The second patient was a 52-year-old man also from India, who had a habit of regular pond bathing. He had a three-year history of nasal rhinosporidiosis and four prior surgeries. During the prior year, he developed not only a verrucous plaque that covered the palmar and medial aspects of his right little finger but also a cutaneous horn in the right popliteal fossa. Microscopic evaluation of both lesions showed multiple thick-walled mature sporangia-releasing endospores. Systemic workup was negative, and the etiology of the skin lesion was suspected to be caused by autoinoculation. There was no improvement after two months of dapsone (100 mg daily) and cotrimazole (double strength twice daily).	[8]
Tuberculosis	Cutaneous tuberculosis is also referred to as lupus vulgaris. At least three patients had been described with lupus vulgaris presenting as a cutaneous horn. The first was a 26-year-old man whose father and uncle died of pulmonary tuberculosis and whose brother also had lupus vulgaris. From 11 years of age, he had a patch of lupus vulgaris on his left cheek that gradually grew. The following year, he developed cutaneous horns presenting as horny growths within the lupus vulgaris. In addition, he also had extensive superficial lupus vulgaris not only on the upper part of his body but also on his right foot. The second patient was presented at the dermatology section of the Royal Society of Medicine. He was a 37-year-old man with a congenital hemangioma involving the right temporal region. At four years of age, he had tuberculosis of the left hip joint. He was treated at the same age for lupus vulgaris of the right cheek and ear. Six years later, at ten years of age, the area affected by lupus vulgaris was excised and a skin-graft applied. For the next 12 years, until age 22 years, the site was treated by ultraviolet light. From 22 to 37 years of age, he received no treatment; after stopping treatment, lupus vulgaris spread over his right cheek. At 35 years of age, two cutaneous horns (measuring 5 and 2.5 cm in height) developed from a common base within the 10-cm area of lupus vulgaris on the right cheek that impinged upon the hemangioma. During the discussion that followed the presentation, one of the doctors in attendance commented that he had seen a patient who had lupus vulgaris-associated cutaneous horns that were more diffusely distributed and appeared as long, thin, horny processes.	[10]
Verruca vulgaris: NOS	Verruca vulgaris is caused by human papilloma virus infection. They are associated with nearly 20% of cutaneous horns. The 22-year-old woman in this report had a 35 mm × 15 mm cutaneous horn on her left fifth toe. Adjacent to the large cutaneous horn, characteristic-appearing verrucous papules were present. Also, condyloma acuminata-related penile cutaneous horns had been observed in men with cutaneous horns at this location.	[1-5]
Verruca vulgaris: HPV-2	A 42-year-old man - a pig butcher in rural China - with HPV-2-associated extensive skin warts on his hands and feet with massive cutaneous horns had been described. His skin warts had been present for 30 years, and the associated horny excrescences had been present for eight years. There were several hundred dense and confluent huge hard cutaneous horns. The horns ranged from 0.5 to 5 cm in diameter and from 0.5 to 21 cm in length. He was successfully cured with multimodality therapy: oral acitretin (40 mg daily) and interferon alfa (5 x 10^6^ units three days per week) for one month; followed by three weeks (five days per week) of electron beam radiation therapy to the hands (total dose of 2,000 cGy with 20 MeV irradiation) and feet (400 cGy each time with 6 MV X-irradiation). Three months after radiotherapy, the hands and feet began to improve; after a year, his hands almost had a normal appearance of the hands, and there had been recovery of his normal daily activity. Another patient, a 50-year-old man, had numerous, confluent, huge HPV-2 wart-like skin lesions on both hands and feet for 13 years. The appearance of small skin horns (ranging from 2- to 3-cm thick) over the wart-like lesions had occurred about one year earlier. Investigation showed that both men’s warts possessed strong promoter activities.	[9]

An uncommon clinical presentation related to human papillomavirus-2 with massive cutaneous horns has also been described in a small number of individuals [1-5,9]. Less frequently, a cutaneous horn has been noted in patients with pox-virus-related molluscum contagiosum [5]. Rarely, infection-associated cutaneous horns have been observed in patients with either leishmaniasis, rhinosporidiosis, or cutaneous tuberculosis [7,8,10].

Malignant neoplasms may be associated with a cutaneous horn (Table 5) [1-5,14-20]. Squamous cell carcinoma, including squamous cell carcinoma in situ (which is also referred to as Bowen’s disease) and keratoacanthomas, is frequently observed at the base of a cutaneous horn. Merkel cell associated with squamous cell carcinoma in situ has also been observed in the cutaneous horn on the face of a 93-year-old woman [16]. Verrucous carcinoma has been observed in patients with a cutaneous horn on the oral commissure and the glans penis [20]. Sebaceous carcinoma-associated cutaneous horn has been noted not only in the periorbital location but also at extraocular sites [19]. Basal cell carcinoma is infrequently (<2%) associated with a cutaneous horn; also, melanoma is seldom associated with a cutaneous horn [3,15].

**Table 5:** Malignant neoplasms associated with cutaneous horns. ^a^Squamous cell carcinoma also includes adenoacanthoma (adenoid squamous cell carcinoma), squamous cell carcinoma in situ (which is also referred to as Bowen’s disease), and keratoacanthoma. HIV, human immunodeficiency virus; Refs, references

Malignancy	Comments	Refs
Basal cell carcinoma	In a study of 230 cutaneous horns, three of the lesions (1.3%) were associated with an underlying basal cell carcinoma. Several other studies have not identified basal cell carcinoma beneath a cutaneous horn.	[3]
Kaposi sarcoma	Kaposi sarcoma had been associated with a cutaneous horn in at least two patients. The first patient was a 79-year-old Italian man with a cutaneous horn on his extensor left forearm that appeared as a firm, protruding, truncated, 1-cm-long, whitish mass; an excisional biopsy demonstrated Kaposi sarcoma in the underlying dermis. A second horn (described as a corn-like mass of 0.5 cm in diameter) was on his left heel; excision also revealed Kaposi sarcoma. The diagnosis was revised to early Kaposi sarcoma after a review of the pathology slides of a diagnosed pyogenic granuloma from his chin (for which the clinical differential diagnosis was verruca vulgaris or pyogenic granuloma) that was removed 32 months prior. The second patient was an HIV-negative 82-year-old Hispanic man with a history of Kaposi sarcoma five years earlier on his left nasal ala (that was treated by excision and radiation), who presented with a cutaneous horn on his right lateral malleolus. The lesion was 17 mm high (with a base diameter of 8 mm) and appeared as a firm, thick, hyperkeratotic horn-like mass. A shave biopsy had been performed showing a dermal spindle cell proliferation which demonstrated human herpesvirus eight slit-like channels and entrapped erythrocytes dissecting through the collagen.	[14]
Malignant melanoma	Malignant melanoma associated with a cutaneous horn had been described in at least three patients. The first patient was a 29-year-old woman with an asymptomatic firm, hyperkeratotic, hyperpigmented conical 1.75 cm × 2.5 cm conical hyperkeratotic protruding mass on her left leg of one-year duration, which would catch in her stockings and occasionally bleed; microscopic examination showed a nodule malignant melanoma with a depth of 7 mm, which extended into the reticular dermis. The second patient was a 70-year-old woman with a 20-year history of a 3.5 cm × 3 cm brown-black maculopapular lesion on her left upper eyelid with a gray-yellow hyperkeratotic giant cutaneous horn formation on the middle. Microscopic examination showed a Clark level IV (Breslow thickness of 8 mm) lentigo maligna melanoma. The third patient was a 73-year-old Japanese woman with 14-mm-high and 9-mm diameter at the base black cutaneous horn on her right cheek. Microscopic examination showed a Clark level IIII (Breslow thickness of 10 mm) verrucous malignant melanoma.	[15]
Merkel cell carcinoma	A 93-year-old woman presented with an enlarging cutaneous horn on the left angle of the mandible of six months duration; excision showed not only a squamous cell carcinoma in situ but also Merkel cell carcinoma in the underlying dermis.	[16]
Paget disease	Paget disease of the breast accounts for 1% to 4% of mammary gland carcinomas - presents as an eczematous patch or plaque involving only the nipple or both the nipple and areola; microscopically, within the epidermis, there is a proliferation of large, pale-staining tumor cells. A 67-year-old white woman developed crusting and oozing of her right breast nipple; over the subsequent five weeks, a cutaneous horn appeared at the site appearing as a hard, asymptomatic mass of thick compact keratinized material. An inadequate superficial shave biopsy did not yield any diagnosis. Three months later, the recurrent and persistent lesion was evaluated after the entire nipple tip was removed. Paget cells were observed in the epidermis. A modified radical mastectomy of the right breast was performed, revealing intraductal carcinoma. No cancer was present in the 28 axillary lymph nodes evaluated.	[17]
Renal cell carcinoma	Cutaneous metastases of renal cell carcinoma can be variable in appearance. A 61-year-old white man presented with an asymptomatic, 2 cm in height and 1.2 cm in diameter at the base, cutaneous horn on the left side of his forehead. His medical history was significant for metastatic renal cell carcinoma (to the renal veins and renal nodes) seven years earlier. He was initially treated with surgery that included a left radical nephrectomy, left adrenalectomy, and splenectomy. Postoperatively, he received 22 monthly treatments of bleomycin and lomustine. Follow-up during the next four to five years was negative for cancer. One month before the appearance of his forehead skin lesion, he had left hip joint pain and a syncopal episode. Roentgenograms of the left hip, the skull, and the chest were performed. The former showed lytic lesions (which were positive on bone scan), and the latter showed apical metastasis of the right lung. Biopsy of the cutaneous horn demonstrated metastatic renal cell carcinoma.	[18]
Sebaceous carcinoma	Sebaceous carcinoma can be ocular and extraocular. It can be a feature of Muir-Torre syndrome with aberration of mismatch repair genes and associated with visceral malignancies. A white man - with a family history of Li-Fraumeni syndrome - in his 50s developed a cutaneous horn of his right upper eyelid. The lesion was dark, crusty, hyperkeratotic, and ulcerated. Microscopic examination showed a sebaceous carcinoma. He died from colon cancer five years later.	[19]
Squamous cell carcinoma^a^	Squamous cell carcinoma is the most associated malignant tumor with cutaneous horns.	[1-5]
Verrucous carcinoma	Verrucous carcinoma is a type of squamous cell carcinoma. Some of these tumors develop into giant cutaneous horns. Verrucous carcinoma has presented as a cutaneous horn not only on the oral commissure and the glans penis but also on non-oral epithelium and extragenital sites. A 74-year-old farmer presented with an 8 cm × 5 cm × 3 cm woody, hard, nontender cutaneous horn over the medial aspect of the right pinna; microscopic examination of the right ear lesion that had been completely excised with a 5 mm margin showed a verrucous carcinoma.	[20]

Kaposi sarcoma has been described in two human immunodeficiency virus-seronegative patients [14]. The 79-year-old Italian man’s lesion was on his extensor left forearm. The 82-year-old man’s Kaposi sarcoma-related cutaneous horn was located on his right lateral malleolus. Metastatic renal cell carcinoma to skin presented as a cutaneous horn on the left side of the forehead in a 61-year-old man [18].

## Conclusions

A cutaneous horn has been observed in association with either a benign or malignant tumor, or an infection, or a skin disorder. Two patients with a cutaneous horn were reported. The first patient was a woman with a recalcitrant verruca vulgaris on her left fifth toe and an associated cutaneous horn on her toe. The second patient was a man with a cutaneous horn associated with an inverted follicular keratosis on his upper lip. The most frequently occurring lesions associated with a cutaneous horn are either actinic keratosis, squamous cell carcinoma, seborrheic keratosis, or verruca vulgaris. Numerous benign tumors have been observed at the base of a cutaneous horn. Rarely, a skin condition, such as discoid lupus erythematosus, palmoplantar keratoderma, psoriasis, or sarcoidosis, has been the cause of a cutaneous horn. Human papillomavirus-related verruca vulgaris is the most frequent infection causing a cutaneous horn. Squamous cell carcinoma is the most common malignant neoplasm associated with a cutaneous horn; basal cell carcinoma, sebaceous carcinoma, verrucous carcinoma, or malignant melanoma are less frequently observed; and Kaposi sarcoma, Merkel cell carcinoma, Paget disease of the breast, or metastatic renal cell carcinoma are rarely noted. In summary, an adequate evaluation of the base of the cutaneous horn is usually required to establish the associated diagnosis.
